# A robust cell culture system for large scale feeder cell-free expansion of human breast epithelial progenitors

**DOI:** 10.1186/s13287-018-0994-y

**Published:** 2018-10-04

**Authors:** Sumanta Chatterjee, Pratima Basak, Edward Buchel, Leigh C. Murphy, Afshin Raouf

**Affiliations:** 1 Department of Immunology, Faculty of Health Sciences, University of Manitoba, Winnipeg, MB R3E 0T5 Canada; 2 Research Institute of Oncology & Hematology, CancerCare Manitoba, Winnipeg, MB R3E 0V9 Canada; 3 Department of Surgery, Section of Plastic Surgery, Faculty of Health Sciences, University of Manitoba, Winnipeg, MB R3A 1M5 Canada; 4 Department of Biochemistry and Medical Genetics, Faculty of Health Sciences, University of Manitoba, Winnipeg, MB R3E 0J9 Canada

## Abstract

**Background:**

Normal human breast epithelial cells are maintained by the proliferation and differentiation of different human breast epithelial progenitors (HBEPs). However, these progenitor subsets can only be obtained at low frequencies, limiting their further characterization. Recently, it was reported that HBEPs can be minimally expanded in Matrigel cocultures with stromal feeder cells. However, variability of generating healthy feeder cells significantly impacts the effective expansion of HBEPs.

**Methods:**

Here, we report a robust feeder cell-free culture system for large-scale expansion of HBEPs in two-dimensional cultures.

**Results:**

Using this cell culture system HBEPs can be exponentially expanded as bulk cultures. Moreover, purified HBEP subtypes can also be separately expanded using our cell culture system. The expanded HBEPs retain their undifferentiated phenotype and form distinct epithelial colonies in colony forming cell assays.

**Conclusions:**

The availability of a culture system enabling the large-scale expansion of HBEPs facilitates their application to screening platforms and other cell-based assays.

**Electronic supplementary material:**

The online version of this article (10.1186/s13287-018-0994-y) contains supplementary material, which is available to authorized users.

## Introduction

The epithelial cells in human breast tissue are mainly of two types; luminal epithelial cells surrounding the lumen, and the basally oriented myoepithelial cells located adjacent to the basement membrane. Collectively, these cells organize themselves to create an elaborate network of bilayered ductal and alveolar structure. These epithelial cells undergo multiple rounds of expansion before terminating into secretory alveolar cells during lactation [1]. The dynamic regenerative capacity of the breast epithelium is maintained by self-renewing breast epithelial stem cells and their downstream progenitors. Evidence for the existence of stem cells in the human breast was first provided by Eirew et al. [2, 3] when they implanted human breast cells admixed with fibroblasts under the kidney capsule of hormone-treated immunodeficient mice and generated breast structures in vivo. The existence of downstream breast epithelial progenitors is supported through in vitro colony forming cell (CFC) assay data [2–5]. These CFC assays enabled the prospective identification and characterization of breast progenitor cells that generate mixed colonies containing both luminal and myoepithelial cells (bipotent progenitors) and cells that generate colonies containing luminal-only cells (luminal progenitors) or myoepithelial-only cells (myoepithelial progenitors). Human breast epithelial progenitors (HBEPs) can prospectively be isolated based on their expression of epithelial cell adhesion molecule (EpCAM) and α6-integrin (CD49f). Bipotent progenitors are enriched in the CD49f^bright^EpCAM^low^ subset and luminal progenitors are enriched in the CD49f^low^EpCAM^bright^ subset of human breast epithelial cells [2, 6, 7]. However, the very small numbers of HBEPs that can be obtained from individual breast reduction samples has limited their further characterization.

Ex vivo conditions for culturing of bulk human epithelial cells obtained from the lungs, colon, and breast tissue in two-dimensional (2D) cultures have been reported [8–13]. However, these cultures require the use of stromal fibroblasts as feeder cells, complicating the study of molecular networks that regulate the proliferation and differentiation of HBEPs. Moreover, the fibroblast passage number, source, and culture conditions can also result in variable expansion of the epithelial cells. More recently, it was demonstrated that HBEPs can be maintained and minimally expanded when placed in 3D Matrigel cocultures with either adipose-derived mesenchymal stem cells (AdMSCs) [14] or stromal fibroblasts [15, 16]. However, such Matrigel cultures can only be carried out on a small scale, yielding insufficient numbers of progenitors to perform large-scale molecular and biochemical assays. Furthermore, these 3D cultures suffer similar challenges as the 2D cultures due to their requirement for the use of feeder cells.

In this report, we describe a feeder cell-free cell culture system that enables the large-scale expansion of purified HBEPs as well as bulk human breast epithelial cells (HBECs). This cell culture system is based on a new growth medium formulation that contains cytokines and small molecule inhibitors, yielding the exponential expansion of the HBEPs which retain their differentiation potential. The availability of a culture system that enables long-term propagation and large-scale expansion of human breast progenitors facilitates their application to screening platforms and other cell-based assays.

## Results

### Interleukin 10 recapitulates AdMSC-induced expansion of HBEPs in organoid cultures

**Fig. 1:**
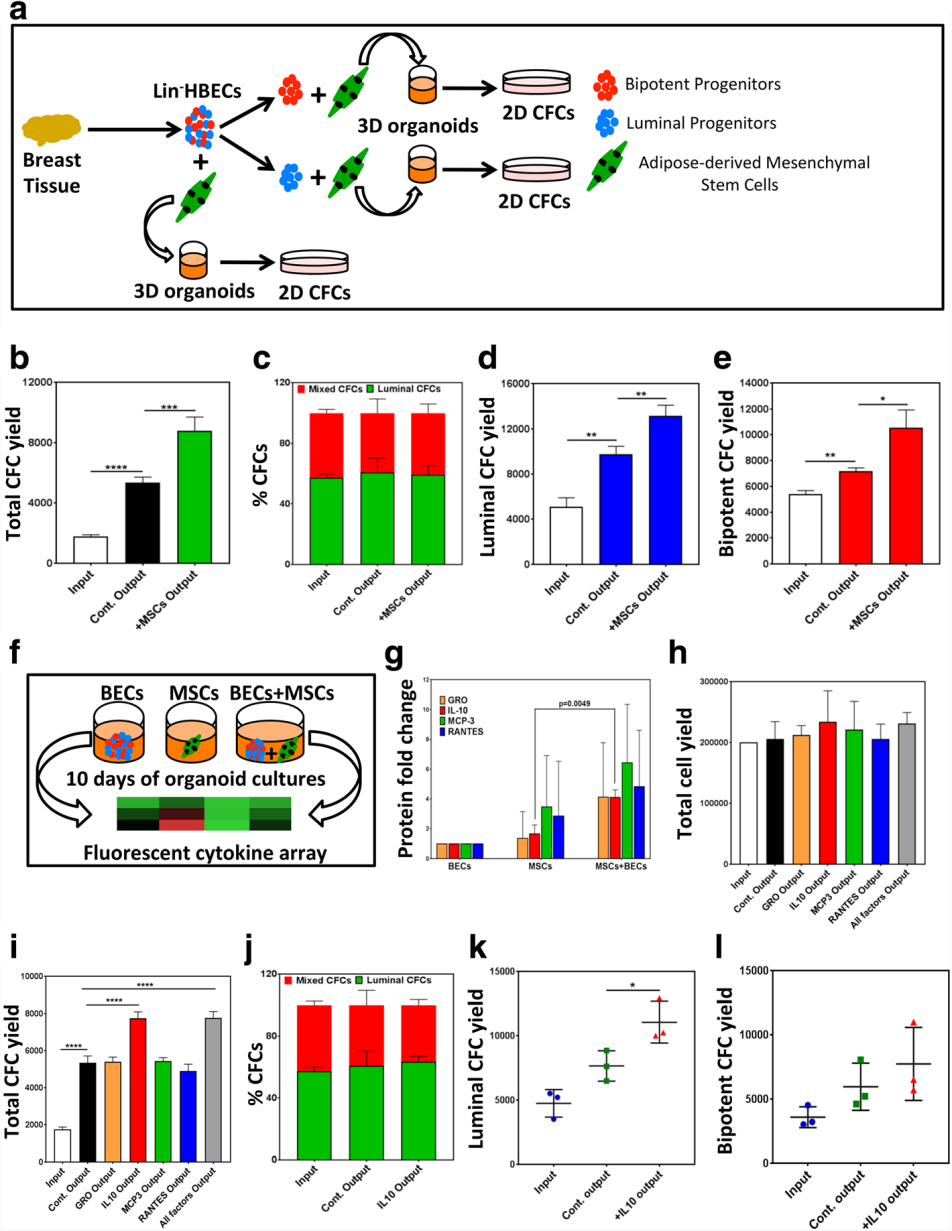
IL-10 recapitulates adipose-derived mesenchymal stem cell-dependent expansion of normal human breast epithelial progenitors. **a** Experimental outline for adipose-derived mesenchymal stem cell (AdMSC)-dependent expansion of human breast epithelial progenitors (HBEPs) in organoid cultures. **b** AdMSC effect on total progenitor (colony forming cell (CFC) numbers) yield in organoid cultures initiated with normal human breast epithelial cells (HBECs). **c** AdMSC effect on frequency of luminal and bipotent CFCs. AdMSC effect on luminal (**d**) and bipotent (**e**) CFC yields in organoid cultures initiated with luminal or bipotent CFC-enriched subsets of HBECs. **f** Experimental outline for cytokine ELISA array performed on conditioned media (CM) obtained from organoid cultures initiated with lineage depleted (Lin^−^) EpCAM^+^ HBECs, AdMSCs, or cocultures of both cell types. **g** ELISA array identified four candidate cytokines upregulated in coculture CM compared to AdMSC-alone CM. All cytokine levels normalized to HBEC CM (= 1). Lin^−^ HBECs placed in organoid cultures and treated with different cytokines and (**h**) average cell numbers and (**i**) CFC yields determined. **j** Effect of recombinant IL-10 on relative yields of luminal and bipotent CFCs. Effect of IL-10 on (**k**) luminal and (**l**) bipotent CFC yields in organoid cultures initiated luminal or bipotent CFC-enriched fractions. All results are represented as means ± SEM from at least three biological replicates. Cont. control, 2D two-dimensional, 3D three-dimensional, GRO growth-regulated oncogene, IL-10, interleukin 10, MCP3 monocyte chemotactic protein 3, MSC mesenchymal stem cell, RANTES regulated on activation, normal T cell expressed and secreted. *p < 0.05, **p < 0.005, ***p < 0.0001, ****p < 0.00001

Figure 1a illustrates the strategy employed to expand purified HBEPs in organoid cultures using AdMSCs as feeders. Lineage-depleted (Lin^−^) bulk human breast epithelial cells (HBECs) or bipotent (Lin^−^EpCAM^low^CD49f^high^) and luminal (Lin^−^EpCAM^high^CD49f^low^) progenitor-enriched fractions were placed in 3D organoid cocultures with AdMSCs (Additional file 1: Figure S1A–C) for 10 days and progenitor numbers were quantified using the CFC assay. HBEPs in the bulk HBECs expanded more significantly in the organoid cocultures with AdMSCs (Fig. 1b) without impacting the distribution of the progenitor subtypes (Fig. 1c) compared to the cultures without AdMSCs. Interestingly, both the bipotent and the luminal progenitor-enriched fractions expanded in organoid cultures (Fig. 1d, e) without AdMSCs; however, the largest progenitor expansion was observed in cocultures with AdMSC (Fig. 1d, e). To test the hypothesis that AdMSC-induced expansion of the HBEPs is due to secreted cytokines and growth factors, the conditioned media (CM) from HBEC-only and AdMSC-only cultures as well as CM from the cocultures were examined using a cytokine enzyme-linked immunosorbent assay (ELISA) array (Additional file 2: Table S1 and Fig. 1f). The array analysis identified GRO, interleukin 10 (IL-10), MCP3, and RANTES as cytokines more significantly present in the coculture CM compared to the HBEC-only or AdMSC-only CM (Fig. 1g). To assess the contribution of each cytokine to the expansion of HBEPs, organoid cultures initiated with Lin^−^bulk HBECs were treated with GRO, IL-10, MCP3, and RANTES and total cell numbers and output progenitor numbers were quantified. None of the cytokines had a significant effect on the total cell numbers in these cultures (Fig. 1h). However, addition of IL-10 significantly expanded HBEPs in the organoid cultures containing bulk HBECs (Additional file 1: Figure S1D and Fig. 1i). Similar to AdMSCs, IL-10 did not alter the distribution of different progenitor subsets in the bulk HBECs (Fig. 1j). More significantly, addition of IL-10 to organoid cultures initiated with progenitor-enriched fractions showed superior expansion compared to the control cultures, although the results were variable (Fig. 1k, l). Surprisingly, neither the organoid-forming potential nor the progenitor marker expression (CD49f and EpCAM) of the bulk HBECs and the sorted bipotent and luminal progenitors were affected by IL-10 treatment (Additional file 1: Figure S1E, F). Next, we examined whether IL-10-expanded HBEPs could be passaged in organoid cultures. While no additional expansion of the HBEPs was observed over three passages, the initially expanded progenitor pool was maintained in these serially passaged 3D cultures (Additional file 1: Figure S1G).

### EpiPro growth medium enables long-term expansion of breast epithelial cells in large-scale 2D cultures

**Fig. 2:**
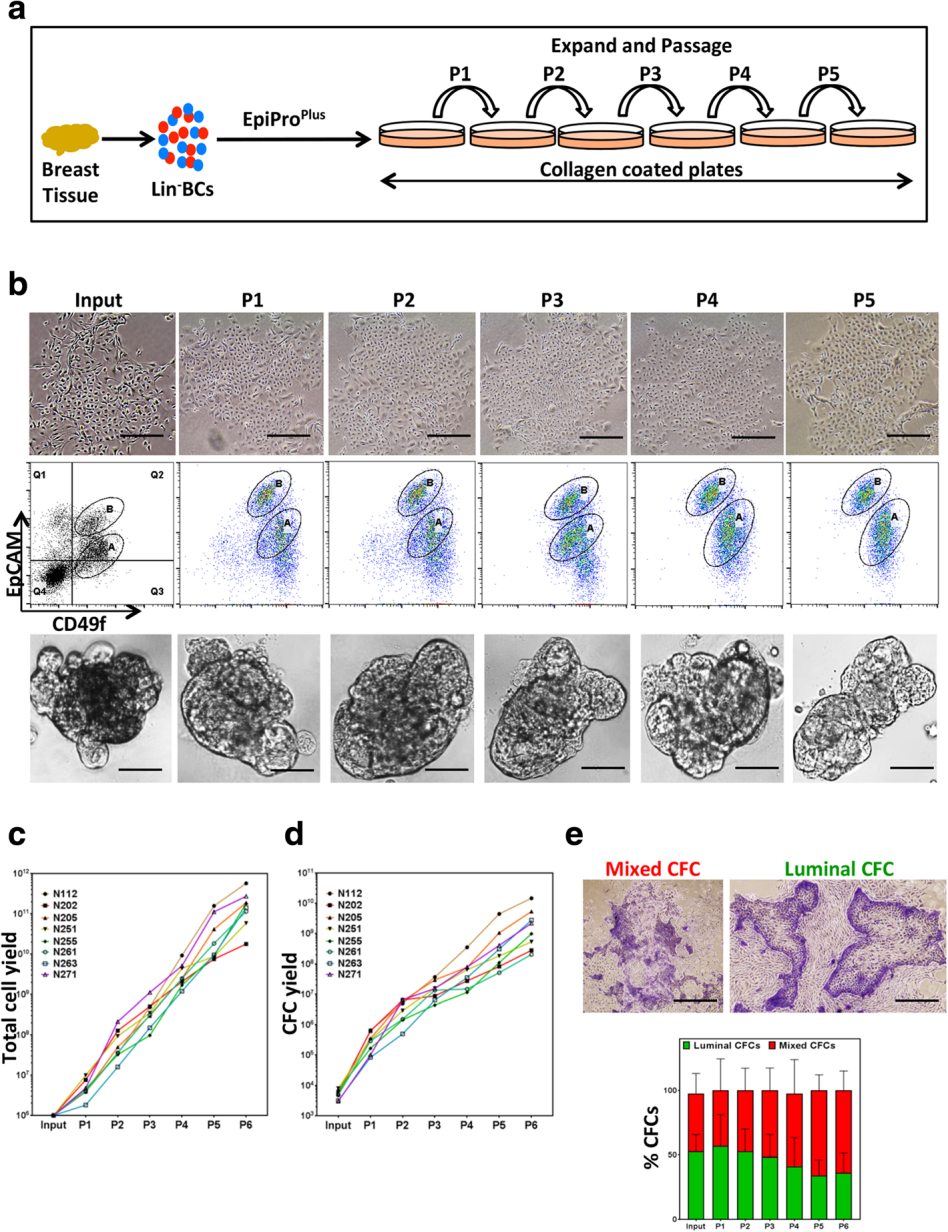
EpiPro^Plus^ growth media enables large-scale expansion of breast epithelial cells in 2D cultures. **a** Experimental outline for ex vivo propagation of primary human breast epithelial cells (HBECs) using EpiPro^Plus^ media (Lin^-^, Lineage-depleted; BC, breast cells). **b** Top panels: representative photomicrographs of HBECs grown in 2D adherent cultures without feeder fibroblasts using EpiPro^Plus^ media over five passages. Middle panels: representative FACS plots depicting CD49f and EpCAM expression in ex vivo expanded HBECs. Lower panels: representative photomicrographs of ex vivo expanded HBECs grown in Matrigels as organoids. Total cell numbers (**c**) and CFC yields (**d**) per passage for different ex vivo expanded cells (generated from eight different reduction mammoplasty samples) are plotted as individual line graphs. **e** Representative colony pictures and progenitor cell frequency (luminal and mixed colony forming cell numbers) are plotted for multiple passages. Results are represented as mean ± SEM from at least three biological replicates. Bars represent 400 μm (**b**) and 1000 μm (**e**). Representative colony pictures and progenitor cell frequencies were plotted for multiple passages. A, B gated cell populations, CFC colony forming cell, EpCAM epithelial cell adhesion molecule, Lin^–^ lineage depleted, P passage

While expansion of HBECs and HBEPs in organoid cultures is interesting, these cultures do not yield the sufficient number of HBEPs required for their further characterization. Epithelial growth factor (EGF)-enriched growth media in combination with feeder fibroblasts have been used in the past to maintain HBECs and HBEPs in 2D adherent cultures. However, this cell culture system is unable to maintain and expand HBEPs over several passages (Additional file 1: Figure S2A, B, red line). More recently, growth medium formulation using TGF-β signaling inhibitors has been used to maintain primary epithelial cells ex vivo in cocultures with feeder fibroblasts [9]. We therefore generated an EGF-enriched growth media formulation that contains both the Rho kinase inhibitor and a TGF-β signaling inhibitor (EpiPro) and examined its effectiveness in maintaining HBECs and expanding HBEPs in 2D adherent cultures without exogenous feeder fibroblasts. Importantly, culturing primary Lin^–^ cells obtained from breast reduction samples in EpiPro media resulted in exponential expansion of HBECs and HBEPs over five passages in 2D tissue culture plates (Additional file 1: Figure S2A, B, green line). Next, we examined whether supplementing EpiPro with IL-10 (EpiPro^Plus^) would further expand HBEPs in these 2D cultures. Figure 2a illustrates the experimental outline for expanding Lin^−^ HBECs on collagen-coated tissue culture plates using EpiPro^Plus^ media. HBECs grown in 2D adherent cultures using EpiPro^Plus^ media can be maintained over five passages while maintaining their epithelial cell phenotype (Fig. 2b). Moreover, these cells maintained expression of bipotent and luminal progenitor cell markers (EpCAM and CD49f; Fig. 2b and Additional file 1: Figure S2E) and readily form 3D organoids in Matrigel (Fig. 2b). However, the double-negative subset of cells (presumably the stromal fibroblasts) and the mature luminal (EpCAM^+^CD49f^−^) cells were not maintainable in the EpiPro^Plus^ media. Over the course of multiple passages, the HBECs showed logarithmic growth and the total HBEP numbers also logarithmically expanded in these samples (Fig. 2c, d). Although not statistically significant, a small decrease in luminal progenitor frequency compared to the bipotent progenitor frequency could be observed in passage 5 and 6 cells (Fig. 2e). Very interestingly, we found that EpiPro^Plus^ was superior to the EpiPro media in expanding HBEC and the HBEP numbers (Additional file 1: Figure S2C, D). In the later passages (4–6), HBECs grown in EpiPro^Plus^ media on average contained 4.7 ± 1.7-fold more HBEPs compared to cells grown in EpiPro media (Additional file 1: Figure S2C, D). It is noteworthy that the interval between passages significantly decreases after the first passage but remains relatively constant thereafter (Additional file 1: Figure S2F).

### EpiPro^Plus^ growth medium enables large-scale expansion of enriched HBEP subsets

**Fig. 3:**
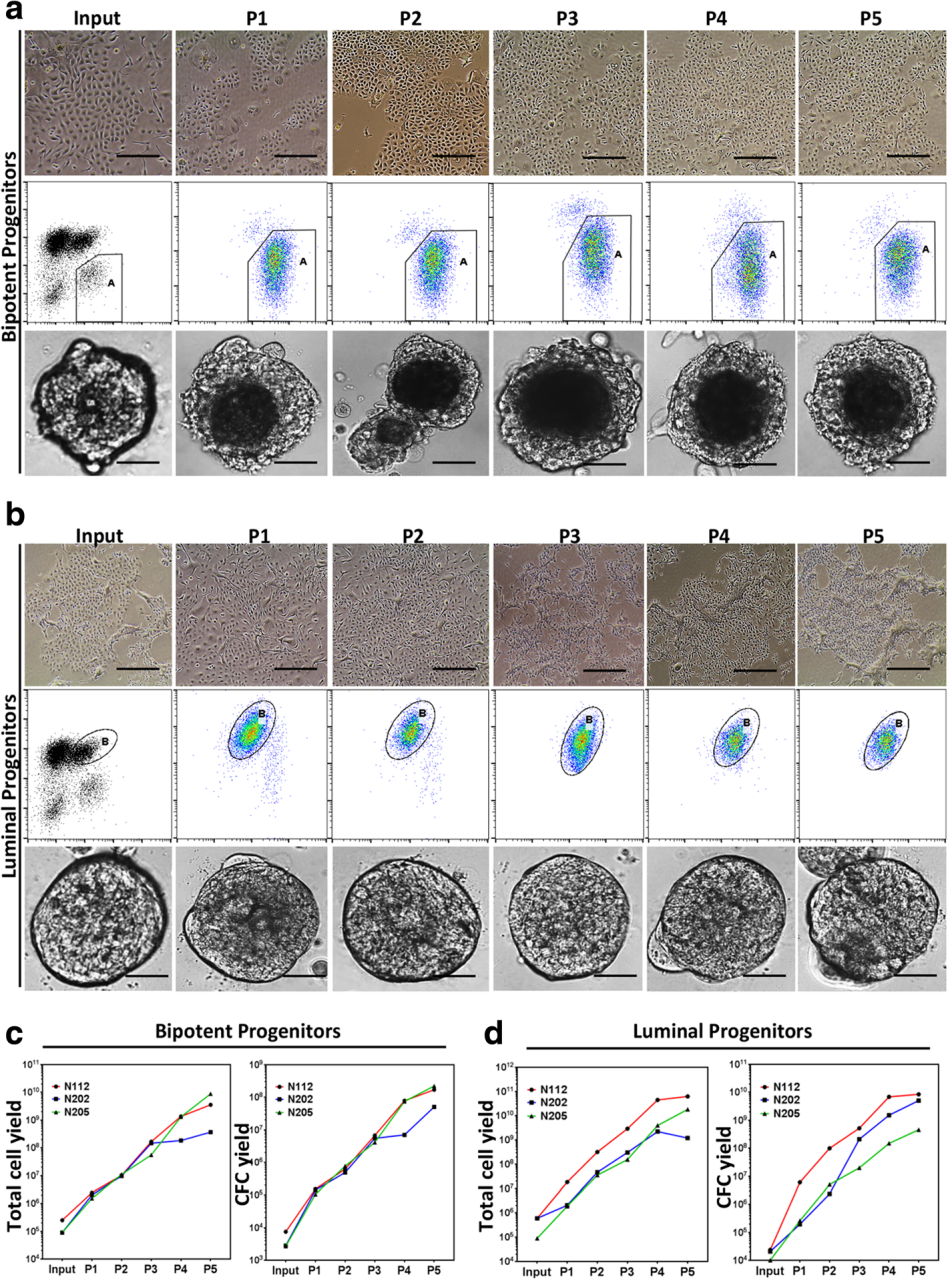
EpiPro^Plus^ medium enables long-term and large-scale expansion of purified breast epithelial progenitors. **a** Top panels: representative photomicrographs of bipotent progenitor-enriched subset of HBECs grown in 2D adherent cultures using EpiPro^Plus^ medium over five passages. Middle panels: representative FACS plots depicting CD49f and EpCAM expression in ex vivo expanded bipotent progenitor (CD49^bright^EpCAM^low^)-enriched fraction. Lower panels: representative photomicrographs of ex vivo expanded bipotent progenitors grown as organoids. **b** Top panels: representative photomicrographs of luminal progenitors (CD49^low^EpCAM^bright^) grown in 2D adherent cultures using EpiPro^Plus^ media over five passages. Middle panels: representative FACS plots depicting CD49f and EpCAM expression in ex vivo expanded luminal progenitor (CD49^low^EpCAM^bright^). Lower panels: representative photomicrographs of 3D organoids grown from ex vivo expanded luminal progenitors. Total cell number and CFC yield per passage calculated and plotted for (**c**) bipotent and (**d**) luminal progenitors for each individual sample. Bars represent 400 μm. Bipotent and luminal progenitors were obtained from three separate mammoplasty samples. A, B gated cell populations, CFC colony forming cell, P passage

**Fig. 4:**
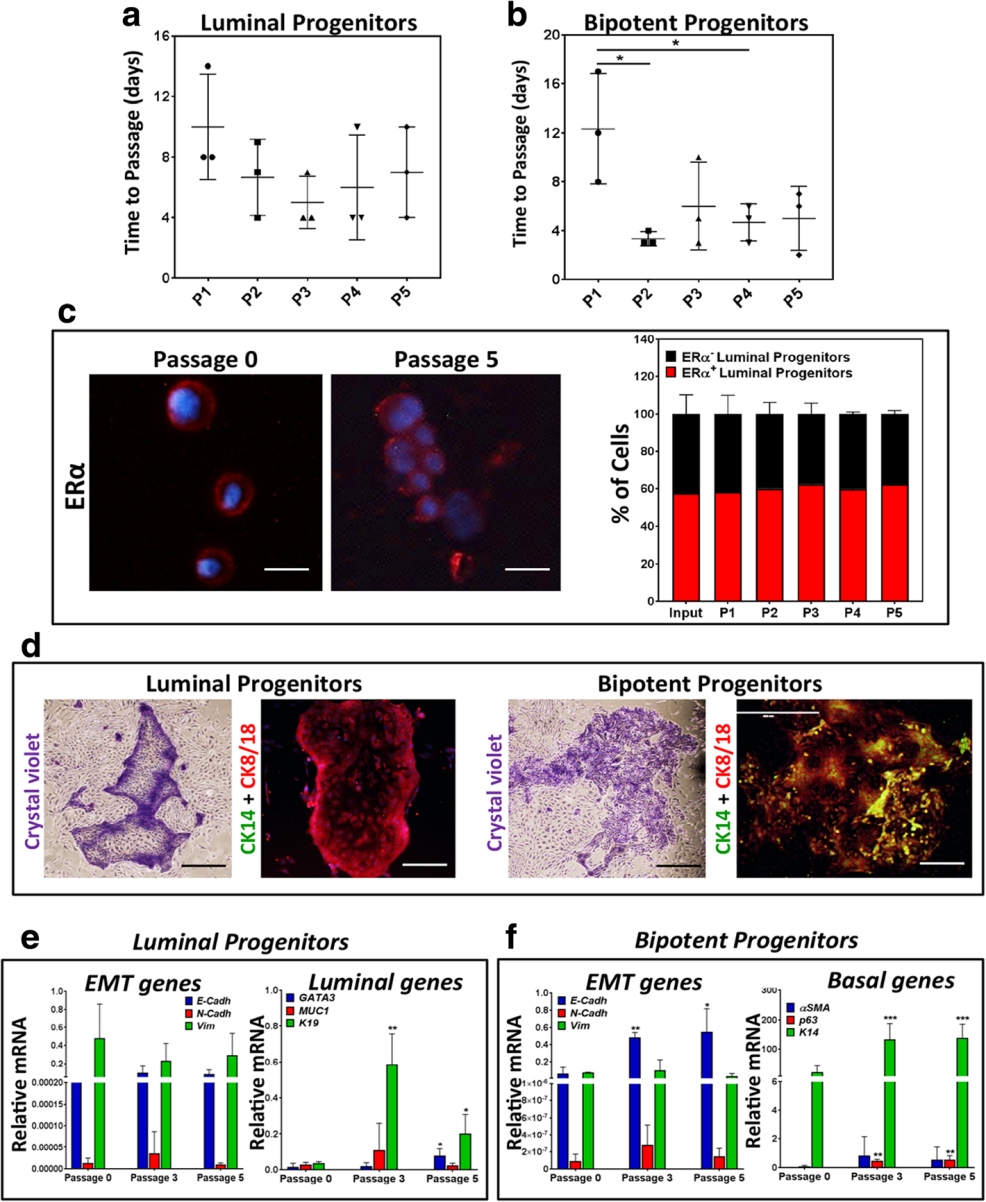
EpiPro^Plus^ medium maintains breast epithelial progenitor cell characteristics in cultures. **a** Average number of days between passages of luminal progenitors (CD49^low^EpCAM^bright^) grown in EpiPro^Plus^ medium. **b** Average number of days between passages of bipotent (CD49^low^EpCAM^bright^) progenitor grown in EpiPro^Plus^ medium. **c** Representative photomicrographs and quantification of ERα^+^ cells in expanded luminal progenitor before ex vivo culturing (passage 0) and after five passages (DAPI nuclear staining in blue, ER staining in red). **d** Representative photomicrographs of colonies from ex vivo expanded luminal and bipotent progenitors immunofluoroscently stained with CK14 and CK8/18 antibodies. Epithelial to mesenchymal transition (EMT) and epithelial specific gene expression in expanded luminal (**e**) and bipotent (**f**) progenitors over subsequent passages. All results are presented as mean ± SEM from three biological replicates. Bars represent 200 μm (**c**), or crystal violet pictures 1000 μm and immunofluorescent pictures 400 μm (**d**). Cadh cadherin, CK cytokeratin, ERα estrogen receptor alpha, P passage, Vim vimentin. *p < 0.05, **p < 0.005, ***p < 0.0001

The bipotent (EpCAM^low^CD49f^high^) and luminal-restricted progenitor (EpCAM^high^CD49f^low^)-enriched subsets of HBECs were placed in collagen-coated culture dishes with EpiPro^Plus^ growth media and followed for up to five passages (Fig. 3a, b). These ex vivo expanded progenitors maintained their cuboidal epithelial cell characteristics throughout the culture period (Fig. 3a, b, upper panels), and their CD49f and EpCAM marker expression profile (Fig. 3a, b, middle panels), and were able to form organoids in 3D Matrigel cultures (Fig. 3a, b, lower panels). More interestingly, both the bipotent and the luminal-restricted progenitors showed exponential expansion in our ex vivo cell culture system (Fig. 3c, d). Cultures initiated with as few as 5 × 10^4^ progenitors, on average, yielded 10^9^ progenitors after five passages (Fig. 3c, d). Luminal progenitors showed no significant difference in the interval between passages, suggesting a similar cell proliferation rate (Fig. 4a). However, for the bipotent progenitors, the passaging time significantly decreased after the first passage, indicating an increase in the cell proliferation rate (Fig. 4b). The ex vivo expanded bipotent and luminal-restricted progenitors maintained their myoepithelial and luminal cell characteristics in our ex vivo cell culture system. The characterization of these expanded progenitor subsets indicated that the ex vivo expanded luminal-restricted progenitors retained high levels (~ 60%) of estrogen receptor alpha (ERα) protein expression (Fig. 4c) and generated colonies containing CK8/18^+^ luminal-only cells in the 2D CFC assay (Fig. 4d). The ex vivo expanded bipotent progenitors generated colonies consisting of both CK8/18^+^ luminal cells and the CK14^+^ myoepithelial cells (Fig. 4d), which is in keeping with their undifferentiated bipotent potential. Finally, gene expression analysis revealed maintenance of the epithelial gene signature as well as the luminal and basal specific gene expression signature in ex vivo expanded luminal and bipotent progenitor cells over multiple passages (Fig. 4e, f and Additional file 3: Table S2).

## Discussion

The study of molecular signals that regulate the proliferation and differentiation of HBEPs requires them to be obtained in large numbers, which is not currently possible. Here we have described two different growth media formulations that enable the large-scale expansion of HBEPs ex vivo. The EpiPro formulation enabled growth and maintenance of primary HBECs and the exponential expansion of HBEPs present in these bulk cultures over six passages. It was recently reported that a growth medium formulation containing small molecule inhibitor of TGF-β signaling along with feeder fibroblasts is able to maintain and expand ERα^+^ primary HBECs while inhibiting ROCK (Rho Kinase) was deemed to prevent expansion of these cells [9]. In that study, however, the number of luminal progenitors was not quantified and therefore it is not possible to ascertain the extent to which, if at all, this culture condition facilitates the expansion of luminal progenitors. Here, however, we report that combining an inhibitor of TGF-β signaling with a ROCKi results in a robust and exponential expansion of luminal progenitors as well as bipotent progenitors. It is possible that the combination of TGF-β signaling and ROCKi results in superior maintenance and expansion of both HBEP subtypes. Moreover, EpiPro medium maintains the primary HBECs and HBEPs ex vivo without the requirement for fibroblasts, thereby eliminating the variabilities associated with different fibroblast culture conditions and the associated secreted factors.

We further developed the EpiPro medium formulation (EpiPro^Plus^) based on our secretome analysis of AdMSC–HBEC cocultures which identified IL-10 as a major cytokine that expands HBEPs in these cocultures. The EpiPro^Plus^ medium on average resulted in an additional 5-fold expansion of the HBEPs. The EpiPro^Plus^ medium formulation robustly expanded both the bipotent and the luminal-restricted progenitors in bulk human breast epithelial cell (HBEC) cultures without altering their ratio and differentiation potential to form distinct colonies (CK8/18^+^ luminal cell-only colonies or CK14^+^CK8/18^+^ basal and luminal cells containing mixed colonies) in functional CFC assays. EpiPro^Plus^ also successfully expanded progenitors in cultures initiated with luminal (EpCAM^high^CD49f^low^) or bipotent (EpCAM^low^CD49f^high^)-enriched epithelial cell fractions. HBEPs propagated in bulk HBEC cultures or as enriched fractions preserved the EpCAM^low^CD49f^high^ bipotent and EpCAM^high^CD49f^low^ luminal progenitor cell phenotypes over multiple passages. Interestingly, however, the expanded HBECs lose CD49^−^EpCAM^−^ stromal and CD49f^−^EpCAM^+^ mature luminal cell populations over time, suggestive of clonal selection of the highly clonogenic progenitor cells over low/non-clonogenic mature cells in these types of culture. EpiPro^Plus^ also helps maintain ERα protein expression in the expanded luminal progenitors. Maintenance of ERα expression is important since it is critical for luminal progenitor cell functions and responsiveness to estrogen signaling [17]. HBEPs expanded using EpiPro^Plus^ maintain basal and luminal gene signatures and do not undergo epithelial to mesenchymal transition. Finally, we show that bipotent and luminal-restricted progenitors form distinct organoids in 3D Matrigels, a phenotype which is preserved in their ex vivo expanded derivative cells. Lastly, we show that bipotent and luminal-restricted progenitors form organoids with distinct morphologies in 3D Matrigels, and these phenotypes are preserved in their ex vivo expanded derivative cells. The availability of an ex vivo culture system that allows large-scale expansion of breast epithelial progenitor cells without affecting their functional properties has significant implications for undertaking basic and translational research studies. Our cell culture system enables the application of screening platforms such as the CRISPR/CAS9 or lentiviral short hairpin libraries and other molecular and biochemical assays to primitive undifferentiated human breast epithelial progenitors for their further characterization and for identification of signaling networks that regulate their proliferation and differentiation as well as investigation and manipulation of factors underlying breast pathology.

### Experimental procedures

#### Tissue sample collection

Normal human breast tissue samples were obtained from healthy individuals undergoing reduction mammoplasty surgeries. Subcutaneous abdominal fat tissue samples were collected from patients undergoing reconstructive breast surgeries. Samples were obtained according to protocols approved by the University of Manitoba’s Research Ethics Board.

#### Tissue dissociation and cell isolation

Our tissue dissociation protocol has been described previously [18]. Briefly, reduction mammoplasty samples were minced with scalpels and dissociated enzymatically and mechanically for 16 hrs in Ham’s F12 and DMEM dissociation media (1:1 vol/vol F12 to DMEM supplemented with 2% wt/vol bovine serum albumin (BSA), 300 U/ml collagenase, 100 U/ml hyaluronidase, 10 ng/ml epidermal growth factor (EGF), 1 mg/ml insulin, and 0.5 mg/ml hydrocortisone (all from Sigma)). The dissociated samples were pelleted and an organoid-rich fraction (by centrifugation at 800 rpm for 1 min), an epithelial–endothelial-enriched fraction (by centrifugation at 1100 rpm for 4 min), and a fibroblast-enriched fraction (by centrifugation at 2400 rpm for 5 min) were obtained by differential centrifugation. Cell fractions were treated with red blood cell lysis buffer (BD Biosciences) as per the manufacturer’s protocol prior to being resuspended and cryopreserved in 6% dimethylsulfoxide (DMSO)-containing fetal bovine serum (FBS)-supplemented medium and stored in liquid nitrogen. Freshly dissociated/thawed epithelial organoid enriched fraction was treated with warm trypsin and incubated at 37 °C for 5–7 min followed by pipette up and down several times. Trypsin was neutralized by adding an equal amount of 2% FBS containing HBSS and mixed well followed by centrifugation at 1200 rpm for 5 min. The resultant cell pellet was then treated with warm dispase and incubated at 37 °C for 5–7 min followed by pipetting up and down several times. Dispase was neutralized by adding an equal amount of 2% FBS containing HBSS and mixed well followed by centrifugation at 1200 rpm for 5 min. Finally, the cell pellet was resuspended in 2% FBS containing HBSS and counted by Bio-Rad cell counter using a trypan blue dye exclusion assay.

#### Cell separation by flow cytometry and analysis

Single-cell suspensions from organoid-enriched fractions obtained from the reduction mammoplasty tissue samples were pre-blocked in 2% FBS-containing Hank’s Balanced Salt Solutions (HBSS) supplemented with 10% human serum for 15 min. Single-cell suspensions were depleted of non-epithelial cells by removing CD31^+^ and CD45^+^ cells (Lin^–^) using a negative EasySep magnetic separation kit (StemCell Tech.) and biotin-conjugated anti-human CD31 (Biolegend) and CD45 (ebioscience) antibodies. Lin^−^ cells were labeled with an allophycocyanin (APC)-conjugated rat antibody to human CD49f (clone GOH3; Biolegend) and unconjugated mouse monoclonal antibody to human EpCAM (clone VU-1D9; StemCell Tech.) followed by phycoerythrin (PE)-conjugated secondary antibody to mouse (Biolegend). Propidium iodide (PI, 1 mg/ml; Sigma) exclusion was used to identify the dead cells. Luminal (PI^−^CD49f^low^EpCAM^bright^) and bipotent (PI^−^CD49f^bright^EpCAM^low^) progenitor-enriched fractions were then isolated at > 99% (for luminal) and 97–99% (for bipotent) purities using a fluorescent activated cell sorter (FACS) (MoFlo XDP; Beckman Coulter). CD49f and EpCAM expression was analyzed using a Guava EasyCyte 8HT Flow cytometer (Millipore).

#### Matrigel cultures

Bulk HBECs (10^5^ cells) or 5 × 10^4^ bipotent or luminal-restricted progenitor subpopulations of HBECs were placed on top of 50 μl polymerized Matrigels (BD Biosciences) in 96-well plates, and incubated for up to 10 days with SF7 [15] medium supplemented with 70 mg/ml bovine pituitary extract. In some experiments, the medium was supplemented with 50 ng/ml IL-10 (StemCell Technologies) or vehicle control PBS (Phosphate Buffered Saline). For other experiments, 10^5^ bulk HBECs or 5 × 10^4^ bipotent or luminal-restricted progenitors were mixed with 1 × 10^5^ or 5 × 10^4^ AdMSCs respectively and then placed on top of polymerized Matrigels. On the indicated days, the Matrigels were dissolved with dispase to recover the cells as per the manufacturer’s protocol. For some other experiments, 2.5 × 10^4^ 2D culture expanded bulk or luminal or bipotent progenitors were plated on top of 50 μl polymerized Matrigel in 96-well plates, and incubated for up to 10 days with SF7 medium supplemented with 70 mg/ml bovine pituitary extract.

#### Colony forming cell assay

The colony forming unit–fibroblast (CFU-F) assay was set up using single cells from passage 2 SVF samples as described previously [14]. Briefly, cells were plated onto tissue culture plates (1000 cells/plate) using complete MesenCult medium (StemCell Technologies) and incubated for 14 days in 5% CO_2_ humidified incubator at 37 °C. Subsequently, the colonies were fixed in methanol and acetone (1:1) solution stained with crystal violet and the fibroblast colony numbers were obtained using an inverted microscope. Breast epithelial cell colony forming cell (CFC) assays were performed as described previously [15]. Briefly, 5000 cells were plated together with mouse 45,000/ml NIH3T3 fibroblasts in 2% FBS-supplemented SF7 media in either six-well tissue culture plates (Cat#657160; CELLSTAR) or 60-mm tissue culture dishes (Cat#628160; CELLSTAR). After 8–10 days, colonies were fixed and enumerated as described. In some cases, colonies were stained with antibodies against cytokeratin 8/18 and cytokeratin 14, and protein expression was detected by fluorescently conjugated antibodies to distinguish colonies containing mixed luminal and myoepithelial cells (positive for both cytokeratins) or luminal cells only (positive for cytokeratin 8/18 only).

#### Propagation of primary breast epithelial progenitors in 2D cultures

Lineage-depleted bulk HBECs were obtained from breast reduction samples and propagated in 2D adherent cultures using the EpiPro growth medium (DMEM-F12 (with 2.5 mM l-glutamine and 15 mM HEPES) base medium supplemented with 2% (v/v) fetal bovine serum, 10 ng/ml human epidermal growth factor, 1 μg/ml insulin, 0.5 μg/ml hydrocortisone, 1% (w/v) bovine serum albumin, 10 ng/ml cholera toxin, 10 μM Y-27632 (Cat#72304; Stem Cell Tech.) and 10 μM SB-431542 (Cat#72234; StemCell Tech.)). More robust and extensive expansion of the HBECs and FACS-sorted progenitors was obtained using EpiPro^Plus^ growth medium (EpiPro base medium supplemented with 50 ng/ml IL-10 (Cat#78024.1; StemCell Tech.)). Briefly, 50,000 luminal and bipotent progenitors (50,000 flow-sorted cells) or 1 × 10^6^ bulk cells were placed in type I collagen-coated (1:30 dilution in PBS as per manufacturer’s protocol, Cat#04902; StemCell Tech.) 60-mm (for progenitors, Cat#628160; CELLSTAR) and 10-cm (for bulk cells, Cat#664160; CELLSTAR) tissue culture plates respectively with EpiPro^plus^ media. From passage 0 onward, cell growth was monitored under the microscope, and when the culture plates reached 70–75% confluence the cells were passaged. For passaging, growth medium was removed and plates were washed 3× with warm phosphate buffered saline PBS and cells were lifted off using warm trypsin (2–5 ml trypsin/plate) and plates were incubated for 5–7 min at 37 °C with occasional agitation. Trypsin was deactivated with an equal volume of HBSS (2% v/v FBS containing Hank’s balanced salt solution). Cells were pelleted via centrifugation at 1200 rpm for 5 min and the cell pellets were resuspended in 2% FBS containing HBSS.

Additional experimental procedures are detailed in Additional file 4.

## Additional files

Additional file 1: **Figure S1** related to Fig. 1. IL-10-dependent expansion of normal breast epithelial progenitors in organoids. (A) Representative photomicrograph of AdMSCs grown in 2D culture. (B) Representative photomicrograph of crystal violet stained CFU-Fs. (C) CFU-F yields from AdMSCs (obtained from 3 independent samples) used for cytokine ELISA array analysis of conditioned media obtained from various organoid cultures (**Table S1**). (D) HBECs in organoid cultures were treated with escalating IL-10 dose for 10 days and progenitor numbers were obtained using CFC assays. The starting (input) progenitor numbers were also obtained via CFC assay and average CFC yields are reported in bar graphs. (E) Representative photomicrographs of Lin- HBECs and luminal or bipotent progenitors grown as organoids with or without recombinant IL-10. (F) Representative FACS plots and bar graphs depicting CD49f and EpCAM expression (population A vs. B) in Lin^−^ HBECs grown as organoids with or without IL-10. (G) CFC yields were measured in organoid cultures of Lin^−^ HBECs with IL-10 over multiple passages. Results represent the mean ± SEM from 3 mammoplasty samples. The bars in microscopic pictures represent 1000μm. **Figure S2** related to Fig. 2. IL-10 plus Y-27632 and SB431542 enhances HBECs expansion efficiency in 2D adherent cultures. Lin^−^ HBECs grown in regular SF7 media with fibroblasts or in EpiPro medium without fibroblasts and total cell yields (A) and CFC yields (B) for each passage are plotted as line graphs. Lin^−^ HBECs propagated in 2D cultures with either EpiPro or EpiPro^Plus^ medium for over 6 passages. Fold changes in (C) total cell yield and (D) CFC yield for each sample are plotted as separate bar graphs. CFC and cell yield for cells cultured in EpiPro medium were made into 1. (E) Shows variations in frequency of bipotent (population A) and luminal (population B) progenitors in Lin^−^ HBECs described in Fig. 2b. (F) Graph shows the average number of days between passages for Lin^−^ HBECs grown in EpiPro^Plus^ medium. All results are the mean ± SEM from 3 replicates. (PDF 590 kb)

Additional file 2 **Table S1.** Cytokine ELISA array data data obtained from conditioned medium obtained from AdMSC-only cultures, HBEC-only cultures, and their respective 10-day cocultures as 3D organoids (XLSX 18 kb)

Additional file 3 **Table S2.** Gene primer sequences. (XLSX 10 kb)

Additional file 4 Supplementary methods. (PDF 93 kb)

## Abbreviations

2D: Two-dimensional
3D: Three-dimensional
AdMSC: Adipose-derived mesenchymal stem cell
CFC: Colony forming cell
CK: Cytokeratin
CM: Conditioned medium
EGF: Epithelial growth factor
ELISA: Enzyme-linked immunosorbent assay
EpCAM: Epithelial cell adhesion molecule
EpiPro: Epithelial progenitor
ERα: Estrogen receptor alpha
HBEC: Human breast epithelial cell
HBEP: Human breast epithelial progenitor
IL-10: Interleukin 10
Lin: Lineage
ROCKi: Rho kinase inhibitor
