# Supplementary figures and images for: A robust cell culture system for large scale feeder cell-free expansion of human breast epithelial progenitors
Source: Stem Cell Res Ther. 2018 Oct 4;9:264. doi: 10.1186/s13287-018-0994-y (PMC6172804; doi:10.1186/s13287-018-0994-y)

**Figure S1**

**A.**

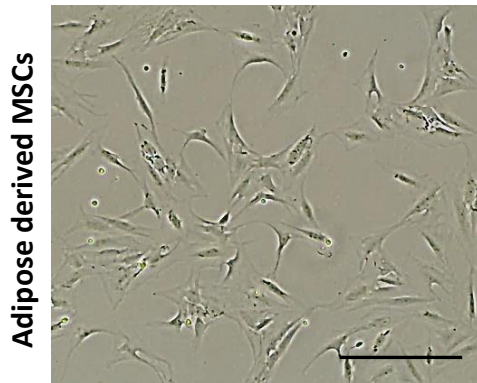

**B.**

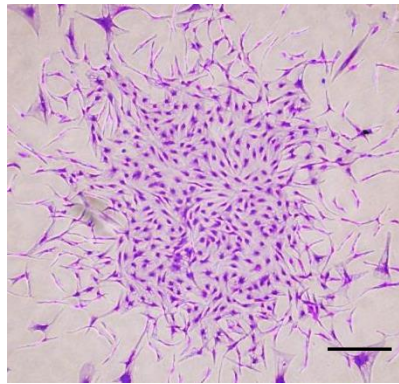

**C.**

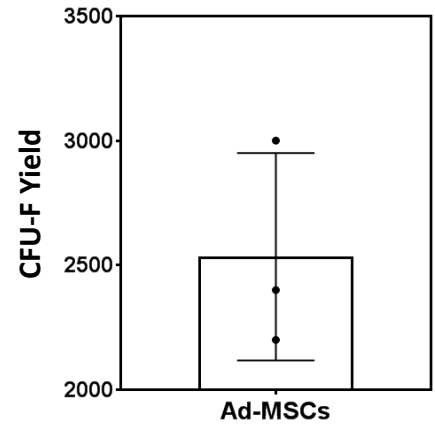

**D.**

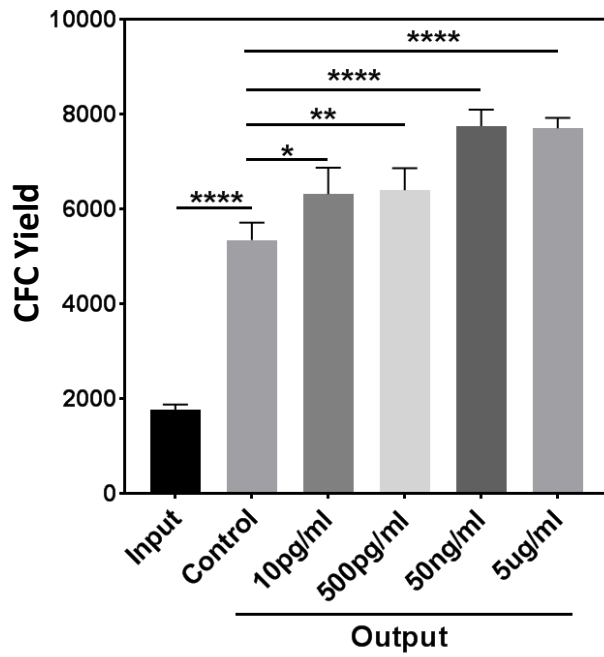

**E.**

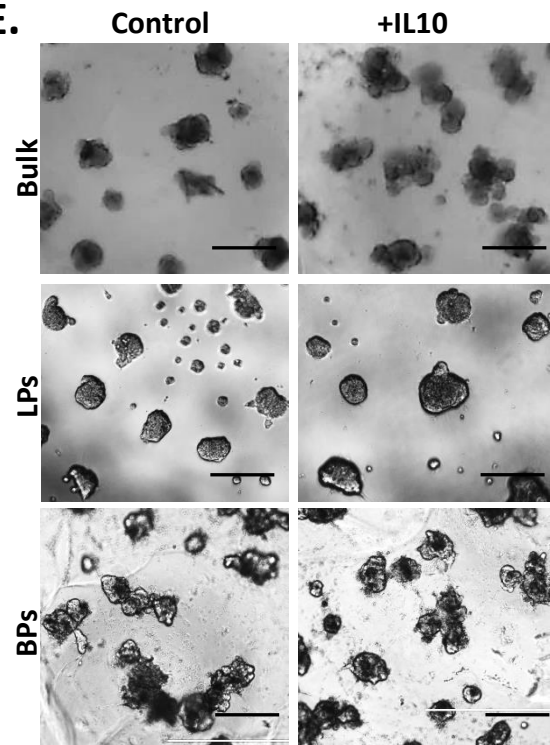

**F.**

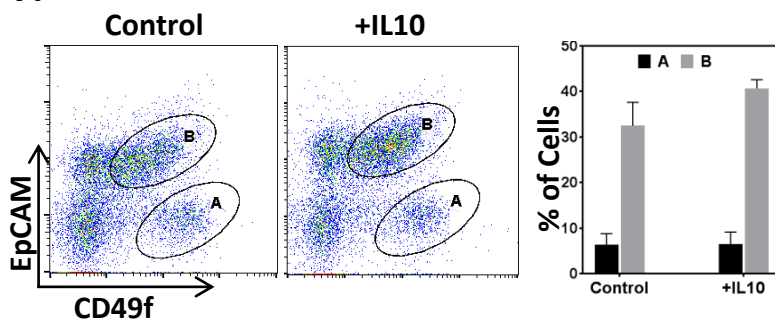

**G.**

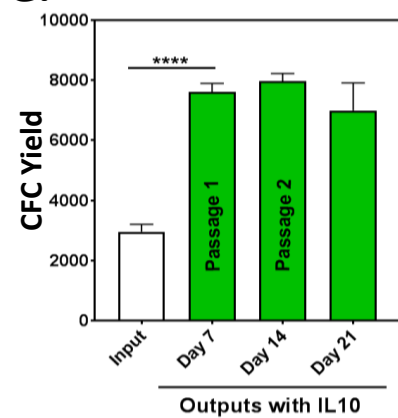

Figure S2

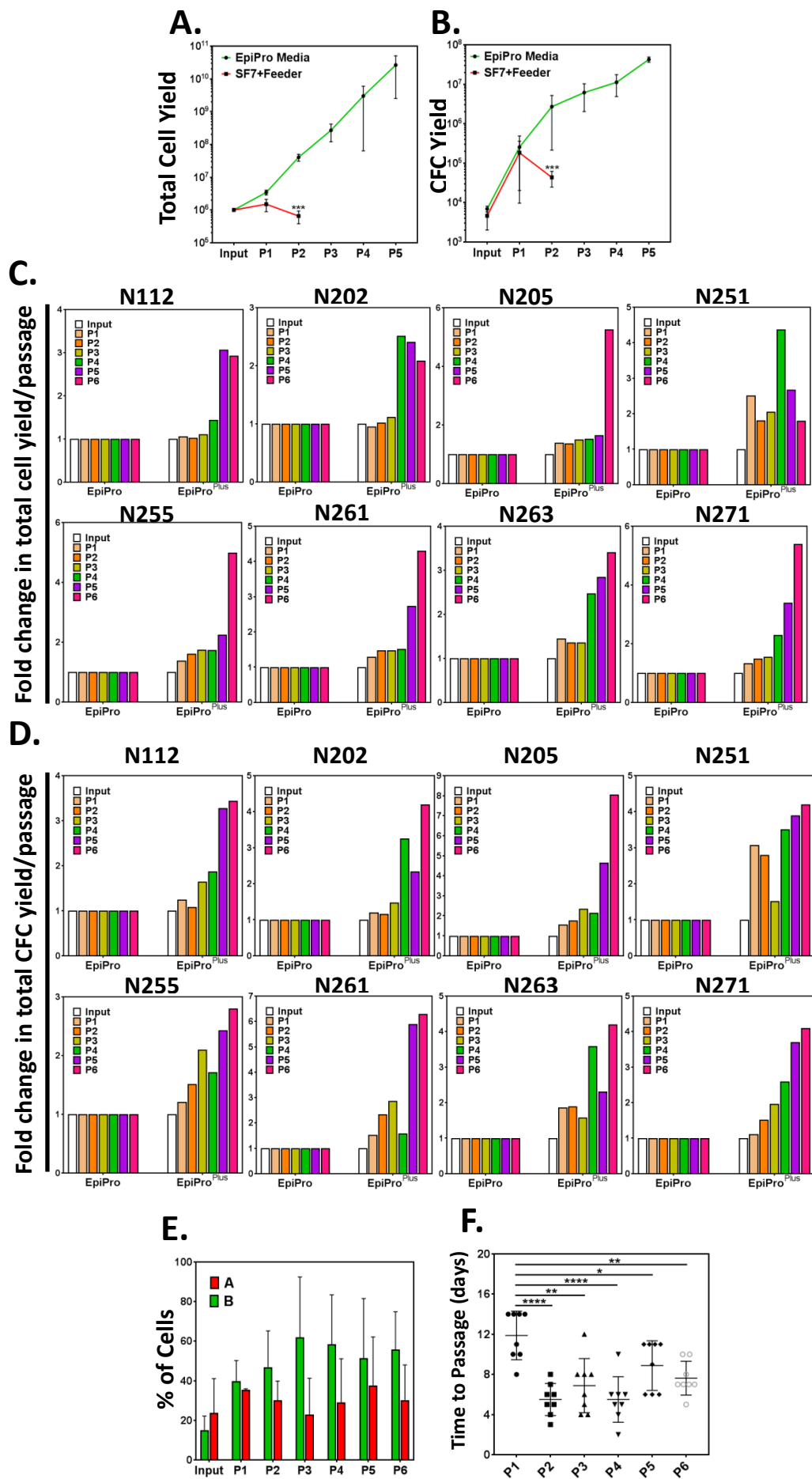

Supplement: Supplementary file 1 — Figure S1 related to Fig. 1. IL-10-dependent expansion of normal breast epithelial progenitors in organoids. (A) Representative photomicrograph of AdMSCs grown in 2D culture. (B) Representative photomicrograph of crystal violet stained CFU-Fs. (C) CFU-F yields from AdMSCs (obtained from 3 independent samples) used for cytokine ELISA array analysis of conditioned media obtained from various organoid cultures (Table S1). (D) HBECs in organoid cultures were treated with escalating IL-10 dose for 10 days and progenitor numbers were obtained using CFC assays. The starting (input) progenitor numbers were also obtained via CFC assay and average CFC yields are reported in bar graphs. (E) Representative photomicrographs of Lin- HBECs and luminal or bipotent progenitors grown as organoids with or without recombinant IL-10. (F) Representative FACS plots and bar graphs depicting CD49f and EpCAM expression (population A vs. B) in Lin− HBECs grown as organoids with or without IL-10. (G) CFC yields were measured in organoid cultures of Lin− HBECs with IL-10 over multiple passages. Results represent the mean ± SEM from 3 mammoplasty samples. The bars in microscopic pictures represent 1000μm. Figure S2 related to Fig. 2. IL-10 plus Y-27632 and SB431542 enhances HBECs expansion efficiency in 2D adherent cultures. Lin− HBECs grown in regular SF7 media with fibroblasts or in EpiPro medium without fibroblasts and total cell yields (A) and CFC yields (B) for each passage are plotted as line graphs. Lin− HBECs propagated in 2D cultures with either EpiPro or EpiProPlus medium for over 6 passages. Fold changes in (C) total cell yield and (D) CFC yield for each sample are plotted as separate bar graphs. CFC and cell yield for cells cultured in EpiPro medium were made into 1. (E) Shows variations in frequency of bipotent (population A) and luminal (population B) progenitors in Lin− HBECs described in Fig. 2b. (F) Graph shows the average number of days between passages for Lin− HBECs grown in [file 13287_2018_994_MOESM1_ESM.pdf]
