# Supplementary material for: A robust cell culture system for large scale feeder cell-free expansion of human breast epithelial progenitors
Source: Stem Cell Res Ther. 2018 Oct 4;9:264. doi: 10.1186/s13287-018-0994-y (PMC6172804; doi:10.1186/s13287-018-0994-y)
Supplement: Supplementary file 4 — Supplementary methods. (PDF 93 kb) [file 13287_2018_994_MOESM4_ESM.pdf]

Supplementary Methods

Adipose-derived mesenchymal stem cell isolation

The stromal vascular fraction (SVF) isolated from abdominal fat tissue was used as source of adipose-derived mesenchymal stem cell (AdMSCs) [1]. Adipose tissue was minced and digested and made into single-cell suspension and treated with red blood cell lysis buffer (Sigma-Aldrich) and cryogenically preserved. For experiments requiring AdMSCs, the SVF cells were thawed and passaged *in vitro* at least twice, as described before to obtain a homogeneous population of AdMSCs.

Immunofluorescent staining

Immunofluorescence staining was performed on the epithelial colonies grown in 2D. Briefly, colonies grown in 6-well culture dishes were fixed in 1:1 vol/vol acetone and methanol solution and pre-blocked in Tris-buffered saline containing 5% wt/vol BSA. Subsequently, plates were stained with unconjugated mouse monoclonal antibody raised against human Cytokeratin 8/18 (Abcam) and rabbit monoclonal antibody raised against human cytokeratin 14 (Abcam). Protein expression was visualized using anti-mouse PE- and anti-rabbit Alexa 488-conjugated secondary antibodies and the cell nuclei visualized by DAPI staining. For immunofluorescence staining of cytospun and air-dried cells on glass slides, cells were permeabilized using ice cold methanol followed by blocking in Tris-buffered saline containing 5% wt/vol BSA. Slides were then incubated with unconjugated rabbit monoclonal antibody raised against human Estrogen Receptor alpha (ERα, SantaCruz Biotechnology) for 1hr at room temperature and protein expression was obtained using anti-rabbit Cy3-conjugated secondary antibody and the nuclei visualized by DAPI staining.

Quantitative real-time PCR

Total RNA was extracted from fresh or cultured cells using the Trizol reagent (Invitrogen) and cDNA was prepared from 1 μg of RNA using the Maxima cDNA synthesis kit (Thermo Fisher) and was used as a template for quantitative real time PCR (CFX Connect 96, Bio-Rad). Transcript expression of specific genes was obtained using gene-specific primers and relative expression level of each transcript was normalized to the *HPRT* transcript level.

Statistical Analysis

The ANOVA and student T-tests were performed using the GraphPad Prism 7.02 program (San Diego, CA).

Supplementary References

1. Chatterjee S, Laliberte M, Blelloch S, Ratanshi I, Safneck J, Buchel E, Raouf A: **Adipose-Derived Stromal Vascular Fraction Differentially Expands Breast Progenitors in Tissue Adjacent to Tumors Compared to Healthy Breast Tissue**. *Plastic and reconstructive surgery* 2015, **136**(4):414e-425e.
